# Expanding the Genetic Framework: Insights into Non-HLA-B27 Contributions to Axial Spondylarthritis

**DOI:** 10.3390/medicina61050793

**Published:** 2025-04-25

**Authors:** Ruxandra-Elena Nagit, Ioana Bratoiu, Corina Cianga, Mariana Pavel-Tanasa, Elena Rezus, Petru Cianga

**Affiliations:** 1 Immunology Department, “Grigore T. Popa” University of Medicine and Pharmacy, 700115 Iași, Romania; ruxandra_nagit@yahoo.com (R.-E.N.); corina.cianga@umfiasi.ro (C.C.); mariana.pavel-tanasa@umfiasi.ro (M.P.-T.); 2 Rheumatology Department, “Grigore T. Popa” University of Medicine and Pharmacy, 700115 Iași, Romania; ioana.bratoiu@umfiasi.ro (I.B.); elena.rezus@umfiasi.ro (E.R.); 3 Rheumatology Clinic, Clinical Rehabilitation Hospital, 14 Pantelimon Halipa Street, 700661 Iasi, Romania; 4 Immunology Laboratory, “St. Spiridon” Clinical Hospital, 700106 Iași, Romania

**Keywords:** ankylosing spondylitis, HLA-B13, HLA-B37, non-HLA-B27 spondylarthritis, pathogenesis, cross-reactivity

## Abstract

*Background and Objectives:* Spondylarthritis is a complex group of inflammatory diseases closely associated with the HLA-B27 antigen. However, the role of non-HLA-B27 alleles in the disease’s pathogenesis has gained significant scholarly attention in recent years. *Case presentation*: This case study presents a 49-year-old male with a history of progressive inflammatory back pain, characterized by morning stiffness and restricted spinal mobility developed over several years. Initially presenting with non-specific symptoms, the patient eventually experienced persistent axial pain and deteriorating functional limitations, which required further evaluation. Radiographic imaging supported the diagnosis of ankylosing spondylitis (AS) by identifying bilateral sacroiliitis. HLA genotyping revealed a negative result for HLA-B27 but positive results for HLA-B13 and HLA-B37. This finding serves as a foundation for exploring alternative genetic factors contributing to spondylarthritis (SpA). HLA-B13 and HLA-B37 exhibit structural and functional similarities to HLA-B27, particularly in their peptide-binding grooves. This resemblance may lead to overlapping peptide repertoires and increased T cell cross-reactivity. Moreover, these alleles belong to overlapping cross-reactive groups (CREGs) and share the Bw4 epitope. This suggests that they may contribute to disease pathogenesis via similar mechanisms, such as molecular mimicry and the dysregulation of natural killer (NK) cell interactions, as observed in HLA-B27. *Conclusions*: This case emphasizes the necessity of expanding diagnostic criteria to incorporate non-HLA-B27 markers, particularly for patients who are HLA-B27-negative. Enhancing our understanding of the roles of alternative genetic markers can improve diagnostic accuracy, enable personalized treatment approaches, and enhance outcomes for the diverse SpA patient population.

## 1. Introduction

Spondylarthritis (SpA) comprises a heterogeneous group of chronic inflammatory diseases that predominantly affect the axial skeleton, entheses, and peripheral joints, often with associated extra-articular manifestations, including uveitis, psoriasis, and inflammatory bowel disease (IBD) [1,2,3,4,5]. Among these conditions, ankylosing spondylitis (AS) is the prototypical form, marked by progressive sacroiliitis and spinal rigidity, leading to substantial functional impairment [6]. Genetic predisposition is crucial in SpA pathogenesis, with the HLA-B*27 allele recognized as the most studied genetic marker associated with this disease. Up to 95% of AS patients test positive for HLA-B*27, underscoring its significance in disease susceptibility and progression [7,8,9].

A substantial percentage of individuals with axial spondylarthritis do not possess the HLA-B*27 allele. This group comprises 37.5% of affected Black individuals [10] and 40% of impacted women [11]. Notably, HLA-B*27-negative patients experience a considerably prolonged diagnostic delay, averaging seven years, which is approximately three years longer than the delay observed in HLA-B*27-positive individuals [12]. Such findings suggest that additional genetic factors may contribute to disease susceptibility and influence the modulation of clinical phenotypes.

In addition to HLA-B*27, several other major histocompatibility complex (MHC) class I molecules have been implicated in the susceptibility to ankylosing spondylitis. Notably, substantial associations have been identified with HLA-B*14, HLA-B*38, HLA-B*40, HLA-B*49, and HLA-B*52 [13]. Furthermore, documented correlations exist between HLA-B*13, HLA-B*47, and HLA-B*51 and the pathogenesis of ankylosing spondylitis [14]. Despite these associations, the precise mechanisms by which these alternative HLA-B alleles contribute to the development of spondylarthritis remain incompletely understood.

Among such non-HLA-B27 alleles, HLA-B13 and HLA-B37 present significant interest due to their structural and immunological properties. HLA-B13 is included in cross-reactive epitope groups (CREGs) that coincide with HLA-B27 and that would imply potential peptide-binding similarities affecting antigen presentation [15,16]. Furthermore, HLA-B13 has been recognized as a genetic factor in AS and psoriatic arthritis (PsA) [17,18] within certain populations. In contrast, the impact of HLA-B37 in SpA determinism is rather under-investigated. Nevertheless, its structural features, notably within the peptide-binding groove, indicate the likelihood of presenting antigenic peptides pertinent to immune activation [19]. Additionally, HLA-B13 and HLA-B37 both exhibit the Bw4 epitope, crucial for natural killer (NK) cell interactions via killer-cell immunoglobulin-like receptors (KIRs) [20].

In this study, we present a patient with advanced ankylosing spondylitis who tested negative for HLA-B27 but tested positive for HLA-B13 and HLA-B37. This case represents, in our opinion, not only a suggestive example of non-HLA-B27 ankylosing spondylitis, but also an important opportunity to investigate the pathogenic mechanisms through which such alternative alleles might contribute to SpA development and progression. Understanding the impact of non-HLA-B*27 alleles on immune activation, antigen presentation, and inflammatory pathways is vital for enhancing our comprehension of SpA’s complex pathogenesis. Furthermore, elucidating the roles of other HLA alleles could have significant implications for refining diagnostic criteria, prognostic evaluations, and therapeutic strategies, especially for the subset of HLA-B27-negative patients who are underrepresented in genetic and clinical research.

## 2. Case Presentation

We present the case of a 49-year-old male residing in an urban setting, with no previously documented chronic conditions. He is a non-smoker and reports no history of alcohol consumption. The patient exhibited chronic inflammatory back pain localized to the lumbar region, accompanied by prolonged morning stiffness. Despite receiving conventional treatment, his symptoms persisted and progressively impaired his daily functioning and overall quality of life.

The patient reported a significant familial predisposition to spondylarthritis, with a sister diagnosed with ankylosing spondylitis and currently undergoing biologic therapy.

**Figure 1:**
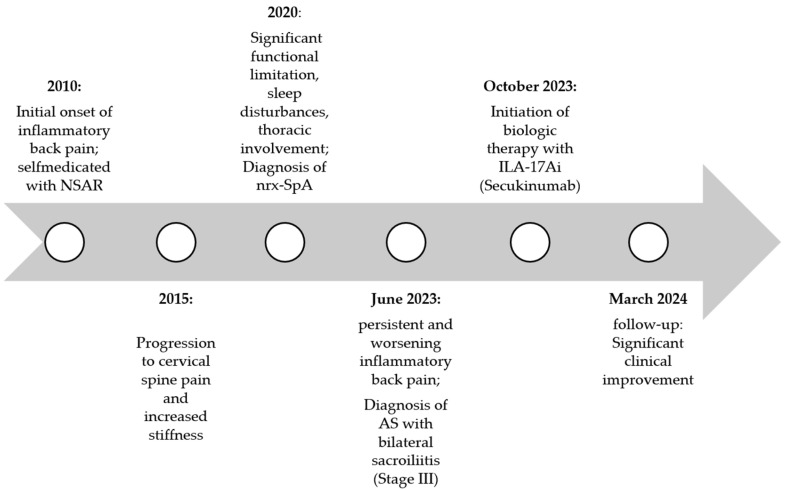
Timeline of the patient’s clinical course.

The patient’s clinical course, depicted in Figure 1, was marked by a gradual but persistent progression of inflammatory symptoms over several years, leading to significant functional impairment and reduced quality of life.

### 2.1. Initial Symptom Onset (2010)

In 2010, the patient began experiencing persistent lower back pain, accompanied by morning stiffness lasting over 30 min. The onset of pain was insidious, characteristically worsening with periods of rest and improving with physical activity, a presentation consistent with inflammatory back pain. At this juncture, the patient did not seek specialized medical attention, nor were any imaging studies or laboratory evaluations conducted. Instead, the patient opted for self-medication utilizing non-steroidal anti-inflammatory drugs (NSAIDs).

### 2.2. Progression and Diagnosis of Non-Radiographic Axial Spondylarthritis (2020)

**Figure 2:**
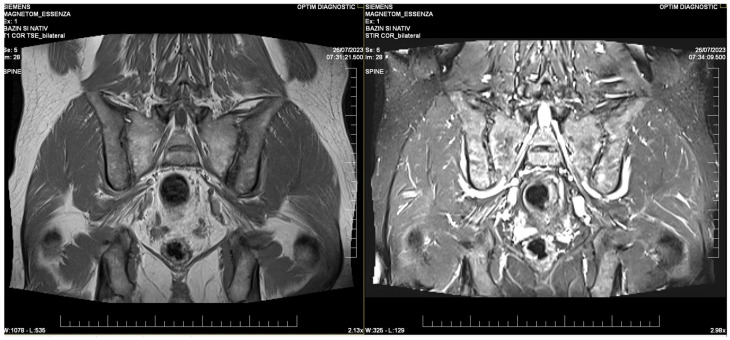
MRI imaging of the sacroiliac joints demonstrates bilateral sacroiliitis. The T1-weighted image (**left**) reveals subchondral sclerosis, joint space narrowing, and erosions, all indicative of chronic structural changes. The STIR sequence (**right**) highlights active inflammation, showcasing bone marrow edema visible as areas of hyperintense signal.

After enduring persistent symptoms for a decade, the patient underwent a rheumatologic evaluation in 2020 due to exacerbating back pain and stiffness. Following a comprehensive clinical assessment, the patient met the Assessment of Spondylarthritis International Society (ASAS) criteria for axial spondylarthritis and was subsequently diagnosed with non-radiographic axial spondylarthritis (nr-axSpA). At this time, no radiographic changes were observed; however, MRI findings revealed typical bilateral sacroiliitis lesions (Figure 2), consistent with the non-radiographic form of the disease. The T1-weighted sequence demonstrated subchondral sclerosis and erosions, while the STIR sequence confirmed active inflammation with bone marrow edema, a hallmark of sacroiliitis. These findings fulfilled the ASAS imaging criterion for axial spondylarthritis, supporting the diagnosis despite the absence of radiographic changes. HLA genotyping yielded a negative result for HLA-B*27; instead, the patient tested positive for HLA-B*13 and HLA-B*37. Given that the patient fulfilled the diagnostic criteria for axial SpA, treatment was initiated in accordance with international guidelines, with continuous NSAID therapy as the recommended first-line approach.

### 2.3. Clinical Deterioration and Persistent Symptoms (June 2023)

In June 2023, the patient was first evaluated in our clinic due to worsening inflammatory pain localized to both the lumbar and cervical spine regions, accompanied by morning stiffness persisting for approximately 40 min. A noticeable decline in functional capacity was observed, characterized by an increasing difficulty in the execution of daily activities. Importantly, there were no reported extra-articular manifestations such as uveitis, psoriasis, or gastrointestinal symptoms during this timeframe.

Laboratory evaluations indicated heightened inflammatory activity, evidenced by an elevated C-reactive protein (CRP) level of 1.9 mg/dL and an erythrocyte sedimentation rate (ESR) of 28 mm/h.

**Figure 3:**
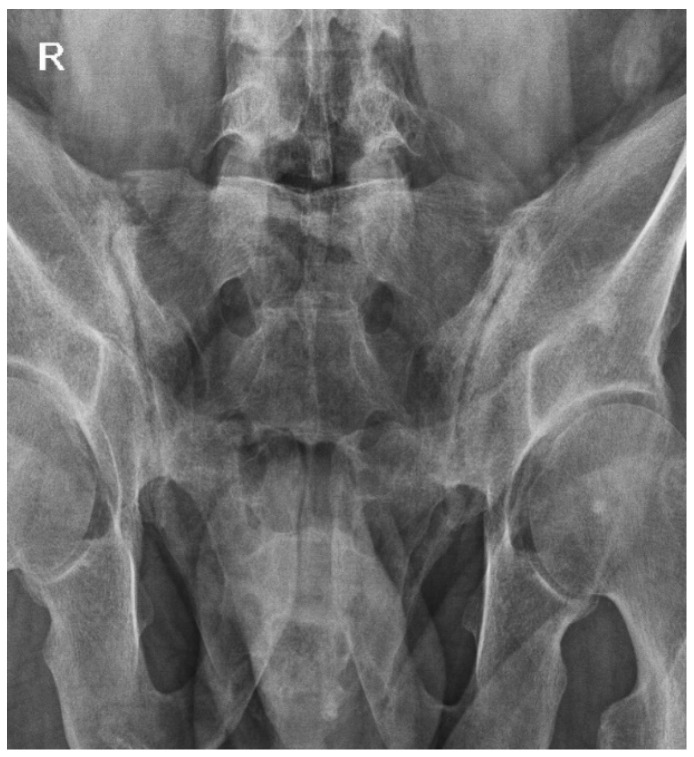
Radiographic imaging of the sacroiliac joints demonstrates bilateral sacroiliitis, classified from stage III to stage IV. Advanced sclerosis is observed bilaterally, accompanied by the presence of superior and inferior ankylosing bridges on the left side, which indicates the progression to advanced ankylosis. On the right side, there are subtle inferior bridging formations, signifying the early stages of ankylotic changes.

At this juncture, conventional radiographic imaging revealed bilateral sacroiliac joint changes consistent with stage III/IV sacroiliitis (Figure 3), thereby indicating the progression of the condition to ankylosing spondylitis.

### 2.4. October 2023

The patient presented with persistent inflammatory back pain localized to both the cervical and lumbar regions, accompanied by a significant reduction in spinal mobility and morning stiffness lasting up to one hour. The physical examination revealed marked limitations in spinal flexibility and mobility, with the Modified Schober Test measuring 10 ± 1.5 cm, indicating severely restricted lumbar flexion. Furthermore, chest expansion was notably reduced, with an inspiratory–expiratory difference of only 1 cm, which reflects thoracic cage stiffness likely attributed to advanced disease progression. The Occiput-to-Wall Distance (OWD) was measured at 30 ± 1.5 cm, thereby supporting the presence of substantial postural and spinal rigidity.

Assessment of sacroiliac joint involvement and entheses yielded positive findings. The patient exhibited a positive sacral compression test, along with positive responses to both Mennell’s maneuver and the shear stress test, consistent with sacroiliac joint inflammation and enthesopathy.

Laboratory investigations corroborated the clinical findings, revealing significantly elevated inflammatory markers. The CRP level was markedly elevated at 4.28 mg/dL (normal < 0.5 mg/dL), while the ESR increased to 41 mm/h, both indicating active systemic inflammation.

Pulmonary function testing via spirometry revealed a severe restrictive lung pattern, consistent with thoracic cage rigidity secondary to advanced axial skeletal involvement.

In light of the progressive nature and high activity of the disease, the patient was commenced on biologic therapy. Considering literature evidence regarding the variable response of HLA-B27-negative patients to tumor necrosis factor (TNF) inhibitors, the decision was made to initiate treatment with an IL-17A inhibitor (Secukinumab), which has demonstrated consistent efficacy in both HLA-B27-positive and HLA-B27-negative patients with spondylarthritis [21].

### 2.5. Current Status and Follow-Up (March–October 2024)

At the time of the most recent evaluation, the patient reported a partial reduction in inflammatory back pain subsequent to the initiation of biologic therapy. There was persistent stiffness; however, an improvement in spinal flexibility was observed.

### 2.6. Allelic Contextualization WSithin the Romanian Population

Given the presence of both HLA-B*13 and HLA-B*37 in this patient, we considered their distribution within the Romanian population as reported by Constantinescu et al. [22]. At the national level, HLA-B*13 and HLA-B*37 were observed at frequencies of 3.64% and 1.16%, respectively. In the Moldovan sub-cohort—reflecting the region from which the patient originates—the reported frequencies were 4.4% for HLA-B*13 and 0.7% for HLA-B*37. This regional variation underscores the importance of interpreting HLA find-ings within their specific population context, particularly in genetically diverse areas such as southeastern Europe.

## 3. Discussion

The presented case of a 49-year-old male diagnosed with ankylosing spondylitis in the absence of HLA-B27 illustrates the diagnostic and therapeutic challenges posed by non-HLA-B27 axial spondylarthritis. While HLA-B27 is not required for the diagnosis of AS, its presence often facilitates earlier recognition, whereas HLA-B27-negative cases may be more prone to delayed identification [23]. In this patient, the diagnosis was confirmed based on clinical presentation, inflammatory back pain, and MRI-confirmed bilateral sacroiliitis, in accordance with established classification criteria.

### 3.1. Expanding the Role of HLA-B Alleles in axSpA

While the role of HLA-B27 in SpA has been extensively studied, the increasing recognition of HLA-B27-negative cases necessitates a broader exploration of alternative HLA associations. The detection of HLA-B13 and HLA-B37 in this patient underscores the need to investigate how other HLA class I molecules may contribute to disease susceptibility and clinical heterogeneity.

Multiple HLA-B variants have emerged as potential contributors to disease susceptibility and variations in clinical phenotype. Among these, one of the most extensively studied is HLA-B*07, although findings are heterogeneous across different populations. Initial observations linked HLA-B*07 to reactive arthritis (ReA) and idiopathic sacroiliitis in American cohorts [24], with subsequent studies confirming associations with axSpA in African American [25] and French (*p* = 0.0008, odds ratio [OR] = 5.91) cohorts [26]. Furthermore, HLA-B*07 has been linked to undifferentiated SpA (USpA) in Tunisian (*p* = 0.043, OR = 5.15) [26], Brazilian (*p* = 0.043, OR = 5.15) [27], and Indian (*p* = 1.14 × 10^7^, OR = 5.348) populations [28]. However, other studies suggest a negative correlation between HLA-B*07 and AS, indicating that its influence on SpA susceptibility may be context-dependent, influenced by genetic and environmental factors unique to each population [13,14,29].

In a comparable manner, the alleles HLA-B*38 and HLA-B*39 have been significantly associated with axSpA and PsA in individuals who are negative for HLA-B27. HLA-B*38 has shown significant associations with AS in European Caucasian populations, with notable findings in Spain (*p* < 0.01, OR = 5.38) [30] and in a cohort primarily composed of White patients from the US (PSOAS and NASC studies), as well as additional cohorts from the UK and Australia (*p* = 0.04, OR = 3.2) [13]. This allele has also been correlated with PsA in Argentine (p = 0.03, OR = 2.95) [31] and Canadian (*p* = 0.04) [32] cohorts, suggesting its role in modulating peripheral and axial inflammatory phenotypes within the SpA spectrum. In parallel, HLA-B*39 has demonstrated a moderate association with AS in Japan (*p* = 0.01) [33] and has been correlated with PsA in Canada (*p* = 0.03) [32].

HLA-B*40 presents an intriguing case of differential disease associations across populations. Early studies (1995) linked it to peripheral SpA [26], while subsequent research (1996) identified its major subtype, HLA-B60, as an independent risk factor for AS [7]. Further studies in Taiwan confirmed associations between HLA-B60 and HLA-B61 with AS in HLA-B27-negative patients [34], with these findings later replicated in Chinese (*p* = 2.54 × 10^4^, OR = 1.65) [13] and European (*p* = 0.001, OR = 0.71) [14] populations.

Another allele that may influence susceptibility to SpA is HLA-B*15. This allele has been identified in cases of SpA and late-onset axial SpA, with research indicating that it follows a clinical trajectory that differs from that of HLA-B27-positive SpA. In various populations, including those from Belgium [35], Mexico [36], Tunisia [37], and Colombia [38], HLA-B*15 has been associated with greater peripheral involvement and a milder disease course, evidenced by significant statistical associations (Mexico: p < 0.01, OR = 3.77; Tunisia: p = 0.002, OR = 18.40; Colombia: p < 0.0001, OR = 20.85). Nonetheless, the effects of this allele appear to vary across populations, as no consistent impact has been noted across all examined groups.

Beyond these, HLA-B*51, primarily associated with Behçet’s disease [39], has also been reported in reactive arthritis (*p* = 0.015, OR = 4.91) [37] and select B27-negative axSpA cohorts [14,40]. Its exact role remains controversial, with some studies supporting a risk association while others propose a neutral or even protective effect [29].

Among less frequently studied alleles, HLA-B*49 has been identified as a moderate risk factor for AS in the same multiethnic cohort that reported associations with HLA-B*38, which included Caucasian, Asian, and African populations (*p* = 0.03, OR = 2.36) [13]. Additionally, within the European subset of this cohort, HLA-B*52 was proposed as a risk allele for AS (*p* = 0.006, OR = 2.85), suggesting a potential population-specific association.

HLA-B*57 exhibits variable associations with SpA; while some studies link its subtype HLA-B57:03 to undifferentiated SpA in African populations (*p* < 0.05, OR = 5.24) [41] and to PsA in Chinese patients (*p* = 5.8 × 10^5^, OR = 20.10) [42], HLA-B*57:01 has been identified as a protective marker for AS within the European cohort of the aforementioned study, which also reported associations with HLA-B*52, HLA-B*49, and HLA-B*38 (*p* = 0.004, OR = 0.45) [13].

### 3.2. HLA-B13 and Its Association with Spondylarthritis

Although historically less studied, HLA-B13 has been sporadically reported to be associated with spondylarthritis, particularly in psoriatic arthritis (PsA). It is well documented for its association with psoriasis, as demonstrated in a meta-analysis conducted in 2013. In 1985, Woodrow and Ilchysyn were among the pioneers to describe the relationship between HLA-B*13 and psoriatic arthritis (*p* = 4.3 × 10^−4^) [17]. Cortes et al. established a moderate association between HLA-B*13:02 and AS with an odds ratio (OR) of 1.43 (*p* = 4.29 × 10^−3^) in a large European cohort [14]. Recently, Šegota et al. assessed the frequency of various HLA-B alleles in subtypes of spondylarthritis, reporting that 13.7% of nr-axSpA patients were positive for HLA-B*13, despite its absence in cases of AS. Furthermore, HLA-B*13 was identified in 25% of patients with radiographic psoriatic arthritis (r-axPsA), suggesting a potential role in disease progression in selected subtypes of spondylarthritis [18]. A robust association of HLA-B*13 with PsA was also reported by Chen et al. in the Chinese population, with an OR of 2.65 (*p* = 4.0 × 10^−6^) [42].

### 3.3. HLA-B37: A Marker of Aggressive Disease Phenotype

In contrast to HLA-B*13, the association between HLA-B*37 and spondylarthritis has been documented with greater consistency in the literature. HLA-B*37 has been identified as a genetic marker correlated with more severe and early-onset forms of PsA and potentially axial SpA.

Alenius et al. reported a statistically significant association between HLA-B*37 and aggressive disease manifestations in PsA within a cohort of patients from northern Sweden. Specifically, HLA-B*37 was linked to early-onset disease, frequently presenting before the age of 40, and was also correlated with increased severity of cutaneous involvement [43].

Furthermore, Šegota et al. discovered that 9.5% of SpA patients possessed the HLA-B*37 allele, exhibiting a statistically significant association (*p* < 0.01) with disease susceptibility and progression [18].

### 3.4. Pathogenic Association Between HLA-B13 and HLA-B37 and Spondylarthritis: A Parallel to B27

HLA-B*13, HLA-B*27, and HLA-B*37 are specific alleles located within the HLA-B locus, which is part of the major histocompatibility complex on chromosome 6 in humans. These alleles encode proteins that are crucial for immune surveillance by presenting intracellularly derived peptide antigens to CD8+ T lymphocytes. This process is essential for the detection and elimination of infected or abnormal cells. Structurally, these proteins consist of a heavy α-chain that is non-covalently bound to β2-microglobulin (β2m). The α-chain features three extracellular domains, designated as α1, α2, and α3, along with a transmembrane domain and a cytoplasmic tail. The α1 and α2 domains create the peptide-binding groove, which is formed by two α-helices positioned over a β-sheet structure. The α3 domain is responsible for interactions with β2m. The peptide-binding groove contains six pockets, labeled A through F, which anchor specific peptide residues. The ends of the peptide-binding groove are conserved across MHC class I proteins, ensuring that the amino-terminal and carboxy-terminal residues of the peptide are anchored in specific positions, primarily within the B and F pockets. The structural conformation of the MHC I proteins allows for the effective presentation of peptides ranging from eight to 10 amino acids in length [44]. Peptide binding occurs through a sequence-dependent mechanism, in which the binding groove demonstrates strong preferences for specific residues at certain positions within the peptide while allowing broader flexibility at others. These critical binding regions, known as anchor motifs, are conserved at key positions, typically the second residue (P2) and the terminal residues (commonly P8, P9, or P10) of the peptide. Consequently, peptide-binding specificity is determined by the variability in both the chelated residues and their anchoring positions, primarily within the B and F pockets [45]. The B pocket, formed by residues 9, 45, 63, 66, 67, 70, and 99, governs the binding of amino acids at the P2 position of the peptide. Conversely, the F pocket, composed of residues 77, 80, 81, and 116, determines the specificity for the peptide’s C-terminal residues [19].

#### 3.4.1. HLA Class I Supertypes

Therefore, HLA class I receptors are characterized by significant polymorphism, which impacts their peptide-binding abilities. In the late 1990s, Sette and colleagues observed that, despite significant variability among these molecules, they display overlapping peptide-binding specificities. This allows for the categorization of these molecules into distinct clusters based on their binding characteristics. They proposed the term “HLA class I supertypes,” which groups HLA alleles into nine distinct categories based on their primary anchor specificity residues within the B and F pockets [46]. This classification has been enhanced in recent years, now including up to 15 supertypes. It has been proposed that, due to the structural and sequence similarities in the peptide-binding grooves of HLAs within the same supertype, peptides that can bind to a specific HLA allele are also likely to be able to bind to other alleles within that supertype [19].

Both HLA-B27 and HLA-B37 molecules belong to the B27 supertype, while HLA-B13 falls under the B44 supertype [19]. The B27 supertype is characterized by a specific structural motif that includes E (glutamic acid) at position 45 and either C (cysteine) or S (serine) at position 67 within the B pocket. Although E at position 45 is not exclusive to the B27 supertype—it is present in some B7 supertype antigens—when it occurs in these molecules, E45 is always paired with F (phenylalanine) or Y (tyrosine) at position 67. Crucially, C at position 67 is unique to the B27 supertype, making it a significant characteristic of HLA-B27 [46]. This uniqueness has been linked to the pathogenesis of spondylarthritis, possibly by promoting free heavy chains (FHCs) formation [47].

Given that HLA variants HLA-B13, HLA-B27, and HLA-B37 fall under overlapping supertypes, it is reasonable to consider that they might share structural similarities in their peptide-binding grooves.

#### 3.4.2. Molecular Mimicry

One of the earliest theories explaining how HLA-B27 contributes to the pathogenesis of SpA is the “arthritogenic peptide” hypothesis. Given the canonical role of class I HLA molecules—processing and presenting intracellular antigens to cytotoxic CD8+ T cells—it has been proposed that HLA-B27 may become a target for autoreactive T cells. This hypothesis is grounded in the concept of molecular mimicry, wherein a peptide derived from a bacterial pathogen structurally resembles a self-peptide. Following infection, CD8+ T cells are activated against the pathogen-derived peptide and may subsequently cross-react with self-peptides presented by HLA-B27 or even with peptides directly derived from HLA-B27 itself at sites of inflammation such as the joints, entheses, skin, intestines, and eyes [48].

The structural similarities in the peptide-binding grooves of HLA-B13, HLA-B27, and HLA-B37 suggest a potential for overlapping peptide repertoires, which may increase the likelihood of T cell cross-reactivity. Moreover, the ability of the peptide-binding grooves of HLA-B13 and HLA-B37 to accommodate peptides similar to those presented by HLA-B27, particularly those derived from intracellular pathogens, underscores their potential to mediate shared immune responses.

#### 3.4.3. Cross-Reactivity

The commercially available anti-HLA-B27 monoclonal antibodies utilized in flow cytometry exhibit cross-reactivity with various other HLA-B molecules due to structural similarities in their epitopes. Levering et al. demonstrated that antibodies specifically designed for the detection of HLA-B27 also cross-react with HLA-B37 and HLA-B13, among other molecules within the CREG 7 group [15]. CREGs consist of HLA antigens sharing analogous serological epitopes based on patterns of antibody cross-reactivity, which arise from structural resemblances in the antigen-binding regions of HLA molecules. Previous research by Cianga et al. (2011) has explored and confirmed these cross-reactivities, particularly those associated with HLA-B7 and HLA-B37 [16]. This evidence prompts an inquiry into whether the structural mimicry observed among these alleles may extend to their pathogenic mechanisms, potentially allowing these molecules to present similar arthritogenic peptides or to elicit comparable immune responses.

HLA-B13 belongs to the cross-reactive epitope group 7 (CREG 7), which also encompasses alleles such as B7, B8, B22 (54, 55, 56), B27, B40 (60, 61), B41, B42, B47, B48, B59, B67, B81, and B82. On the other hand, B37 is part of B12 CREG, which also includes B12 (44, 45), B13, B21 (49, 50), B40 (60, 61), and B47 (https://www.ufrgs.br/imunovet/molecular_immunology/hla.html#CREG, accessed on 25 February 2025). Notable is the fact that HLA-B13 is classified under both B7 and B12 CREGs, while B27 and B37 both share the public epitope Bw4 (Table 1).

The overlapping classification of HLA-B13, -B27, and -B37 within the B7 and B12 CREGs, along with their shared expression of the Bw4 epitope, suggests an enhanced capacity for peptide presentation. This broadened repertoire increases the likelihood of molecular mimicry. This mechanism, well established for HLA-B27, could also be relevant for HLA-B13 and HLA-B37, expanding the range of microbial triggers capable of inducing or sustaining chronic inflammation.

The Bw4 epitope is a key regulator of immune responses through its interaction with killer-cell immunoglobulin-like receptors (KIRs), which are predominantly expressed on natural killer (NK) cells [20]. KIRs play a crucial role in immune surveillance by detecting HLA class I molecules and modulating NK cell activity. Structurally, KIRs consist of either two or three immunoglobulin-like domains and are classified based on the length of their cytoplasmic tails: receptors with long tails (2DL, 3DL) function as inhibitory receptors, while those with short tails (2DS, 3DS) act as activating receptors. Inhibitory KIRs selectively bind to specific HLA class I ligands with varying affinities. Notably, HLA-B alleles carrying the Bw4 motif are primary ligands for the inhibitory receptor KIR3DL1, establishing one of the most potent and biologically significant inhibitory interactions within the immune system [53]. Although KIRs share structural similarities with T cell receptors (TCRs) in their ability to recognize peptides presented by class I HLA molecules, their functional roles diverge significantly. While TCR engagement typically activates T cells, the interaction of inhibitory KIRs with their HLA ligands generally suppresses NK cell activity [54].

Among the KIRs, KIR3DL2, an inhibitory receptor expressed on both NK cells and CD4+ T lymphocytes, plays a critical role in the pathogenesis of ankylosing spondylitis. A hallmark pathogenic feature of HLA-B27 in SpA is its propensity to form FHCs on the cell surface [55]. This process results from the molecule’s unique structural properties, including its ability to bind unusually long peptides and its slow folding kinetics, which increase the risk of misfolding and homodimer formation [56]. Additionally, HLA-B27 shows reduced dependence on tapasin for peptide loading, allowing improperly folded complexes to escape the endoplasmic reticulum (ER). Once at the cell surface, these complexes can dissociate, leaving unstable FHCs [47]. These FHCs are recognized by receptors like KIR3DL2 on NK cells and CD4+ T cells, enhancing the differentiation and survival of KIR3DL2+ CD4+ T cells, subsequently enhancing the production of pro-inflammatory cytokines, including IL-17, IL-23, TNF-α, and IFN-γ [57].

Moreover, the HLA-B27:05 subtype, strongly associated with AS, exhibits a heightened tendency to form FHC homodimers compared to the non-pathogenic B27:09 variant [57,58,59]. In this light, aberrant interactions between other Bw4-expressing HLA alleles, such as HLA-B13 and -B37, and KIRs can arguably disrupt the delicate balance between immune activation and inhibition, which may lead to inappropriate NK cell activation or inhibition, fostering chronic inflammation and tissue damage.

Understanding the impact of non-HLA-B27 alleles on immune activation, antigen presentation, and inflammatory pathways is vital for enhancing our comprehension of SpA’s complex pathogenesis. Furthermore, elucidating the roles of other HLA alleles could have significant implications for refining diagnostic criteria, prognostic evaluations, and therapeutic strategies, especially for the subset of HLA-B27-negative patients who are underrepresented in genetic and clinical research.

At present, there are no universally accepted genetic screening protocols for HLA-B27-negative SpA, and diagnosis continues to depend largely on clinical assessment and imaging techniques. However, active research into polygenic risk scoring and genome-wide association studies (GWASs) is underway, which may enhance risk stratification and promote earlier identification in the future.

From a therapeutic standpoint, management guidelines for HLA-B27-negative patients align closely with those for HLA-B27-positive individuals. In these cases, the selection of biologic treatment options is primarily guided by the patient’s clinical phenotype, rather than their genetic status. However, emerging evidence indicates that treatment responses may differ between these two groups. For instance, several studies suggest that TNF inhibitors may demonstrate reduced effectiveness in HLA-B27-negative patients. Conversely, IL-17 inhibitors have shown efficacy across all HLA subgroups, highlighting a potential area for therapeutic optimization [21]. Further investigation into the role of non-HLA-B27 alleles in spondylarthritis could elucidate their influence on disease susceptibility and treatment response. Such insights could ultimately pave the way for a more refined, genetics-informed approach to both diagnosis and therapy.

## 4. Conclusions

The management of HLA-B27-negative spondylarthritis presents unique challenges, highlighting the importance of exploring alternative genetic and immunologic contributions to disease pathogenesis and treatment response.

In the case presented, HLA-B13 and HLA-B37 serve as examples of how non-HLA-B27 antigens may contribute to SpA pathogenesis. These molecules share structural similarities with HLA-B27, particularly in their peptide-binding grooves, resulting in possible overlapping peptide repertoires. Such shared features enhance T cell cross-reactivity, potentially driving chronic inflammation and autoimmune responses. Furthermore, HLA-B13, HLA-B27, and HLA-B37 are classified within overlapping cross-reactive groups and share the Bw4 epitope, which may influence disease progression through mechanisms such as molecular mimicry, altered NK cell and T cell regulation via Bw4-KIR interactions, and the presentation of immunogenic peptides.

A critical consideration here is the concurrent presence of the HLA-B13 and HLA-B37 antigens, which complicates the determination of their relative contributions to disease pathogenesis. Statistically, HLA-B37 demonstrates a more robust association with spondylarthritis; however, it is plausible that the disease may arise from a synergistic interplay between the two alleles. Their simultaneous expression could potentially modulate T cell activation, enhance antigen presentation, or affect immune regulation in a manner that each allele may not independently achieve.

While the pathogenic role of HLA-B13 and HLA-B37 serves as a starting point, it opens the door to a more comprehensive investigation of additional HLA variants that may influence disease susceptibility, clinical presentation, and treatment response.

This broader perspective challenges the traditional focus on HLA-B27 and emphasizes the need to expand diagnostic and therapeutic frameworks to include other HLA alleles. By integrating these insights into clinical practice, we can refine diagnostic accuracy, personalize treatments, and improve outcomes for the diverse population of patients with SpA.

## Figures and Tables

**Table 1:** HLA Cross-Reactive Groups (CREGs) and Their Antigen Specificities.

CREG Group	Included HLA Antigen Specificities
1C	A1, 3, 9 (23, 24), 11, 29, 30, 31, 36, 80
10C	A10 (25, 26, 34, 66), 11, 28 (68, 69), 32, 33, 43, 74
2C	A2, 9 (23, 24), 28 (68, 69), B17 (57, 58)
5C	B5 (51, 52), 15 (62, 63, 75, 76, 77), 17 (57, 58), 18, 21 (49, 50), 35, 46, 53, 70 (71, 72), 73, 78
7C	B7, 8, 13, 22 (54, 55, 56), 27, 40 (60, 61), 41, 42, 47, 48, 59, 67, 81, 82
8C	B8, 14 (64, 65), 16 (38, 39), 18, 59, 67
12C	B12 (44, 45), 13, 21 (49, 50), 37, 40 (60, 61), 41, 47
Bw4	A23, 24, 25, 32, B13, 27, 37, 38, 44, 47, 49, 51, 52, 53, 57, 58, 59, 63, 77
Bw6	B7, 8, 18, 35, 39, 41, 42, 45, 46, 48, 50, 54, 55, 56, 60, 61, 62, 64, 65, 67, 71, 72, 73, 75, 76, 78, 81, 82

Adapted from Universidade Federal do Rio Grande do Sul (https://www.ufrgs.br/imunovet/molecular_immunology/hla.html#CREG, accessed on 25 February 2025), based on data from Rodey & Fuller (1987) [49], Rodey (1994) [50], Thompson (1998) [51], and Duquesnoy (2003) [52].

## Data Availability

The original contributions presented in this study are included in the article.

## Abbreviations

The following abbreviations are used in this manuscript:

AS	ankylosing spondylitis
ASAS	Assessment of Spondylarthritis International Society
β2m	β2-microglobulin
CI	confidence interval
CREG	cross-reactive group
CRP	C-reactive protein
ESR	erythrocyte sedimentation rate
FHC	free heavy chain
HLA	human leucocyte antigen
IBD	inflammatory bowel disease
ILA-17Ai	interleukin 17A inhibitor
KIR	killer-cell immunoglobulin-like receptor
MHC	major histocompatibility complex
MRI	magnetic resonance imaging
NGS	next-generation sequencing
NK	natural killer cell
NRXSPA	non-radiographic spondylarthritis
NSAIDs	non-steroidal anti-inflammatory drugs
OWD	Occiput-to-Wall Distance
OR	odds ratio
PsA	psoriatic arthritis
ReA	reactive arthritis
RR	relative risk
SSP-PCR	single specific primer-polymerase chain reaction
TCR	T cell receptor
TNF	tumor necrosis factor
USpA	undifferentiated spondylarthritis
